# Supplementary material for: An exceptionally preserved Sphenodon-like sphenodontian reveals deep time conservation of the tuatara skeleton and ontogeny
Source: Commun Biol. 2022 Mar 3;5:195. doi: 10.1038/s42003-022-03144-y (PMC8894340; doi:10.1038/s42003-022-03144-y)
Supplement: Supplementary file 2 — Supplementary Information [file 42003_2022_3144_MOESM2_ESM.pdf]

Supplementary Information

An exceptionally preserved *Sphenodon*-like sphenodontian reveals deep time conservation of the tuatara skeleton and ontogeny

Tiago R. Simões, Grace Kinney-Broderick and Stephanie E. Pierce

Contents:

Supplementary Figures (p. 2-7)
Supplementary Table (p. 8-10)
Supplementary References (p. 10)

Supplementary Figures and tables

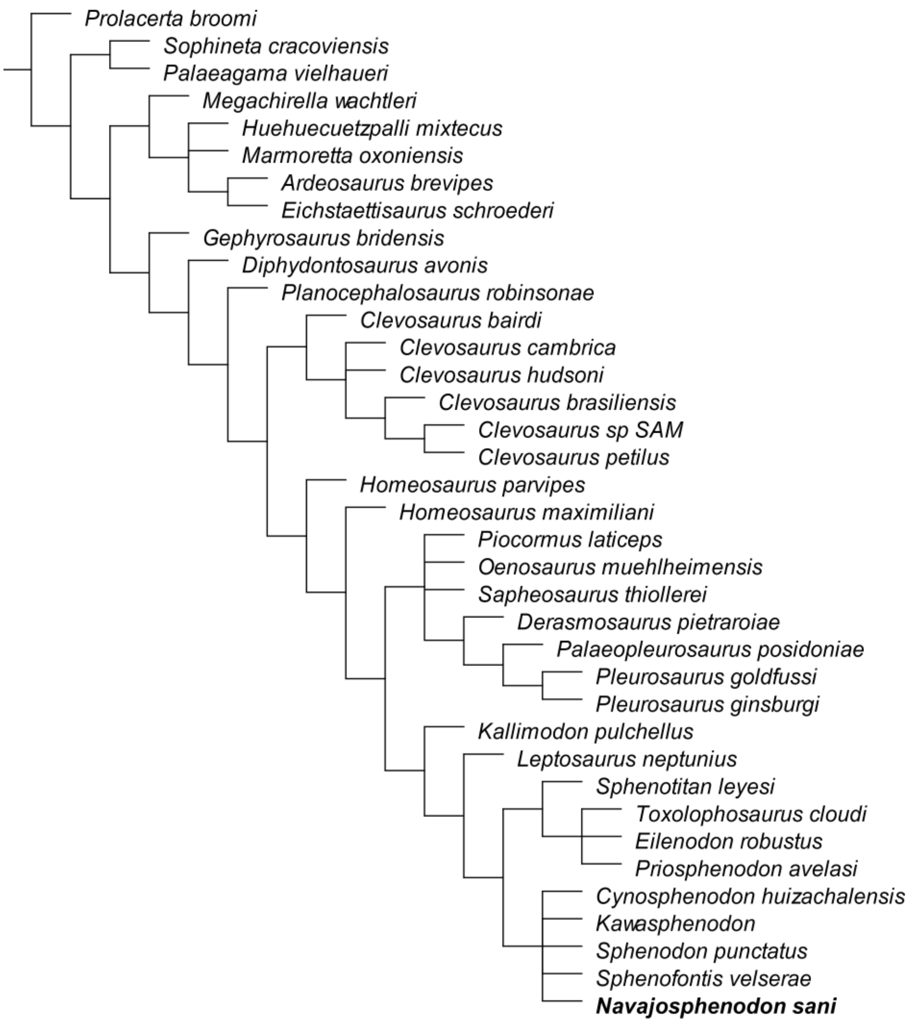

Suppl. Fig. 1. Strict consensus tree from 108 most parsimonious trees (282 steps each) from the equal-weights maximum parsimony analysis.

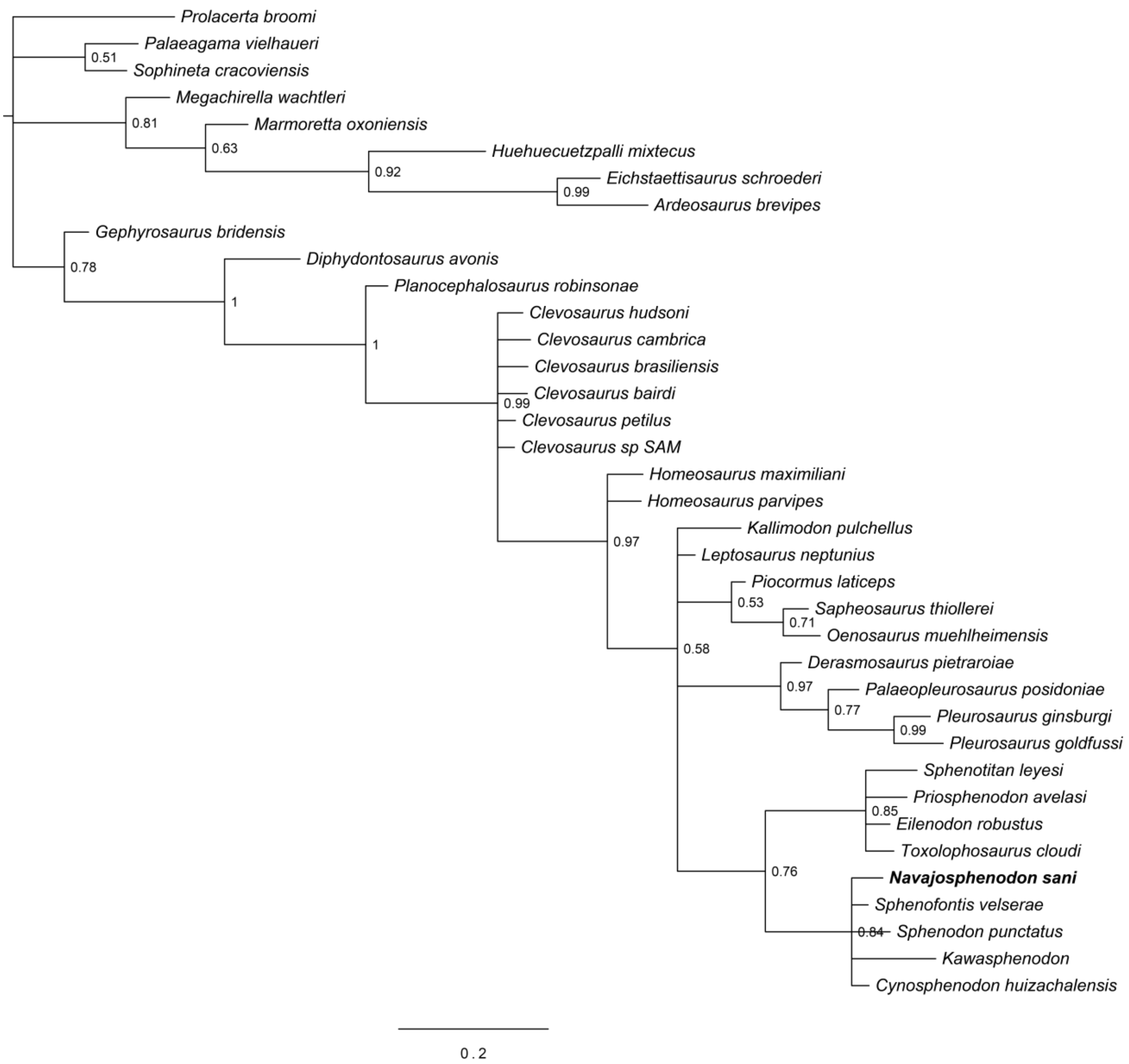

Suppl. Fig. 2. Majority rule consensus tree from the non-clock Bayesian inference analysis. Node values indicate posterior probabilities.

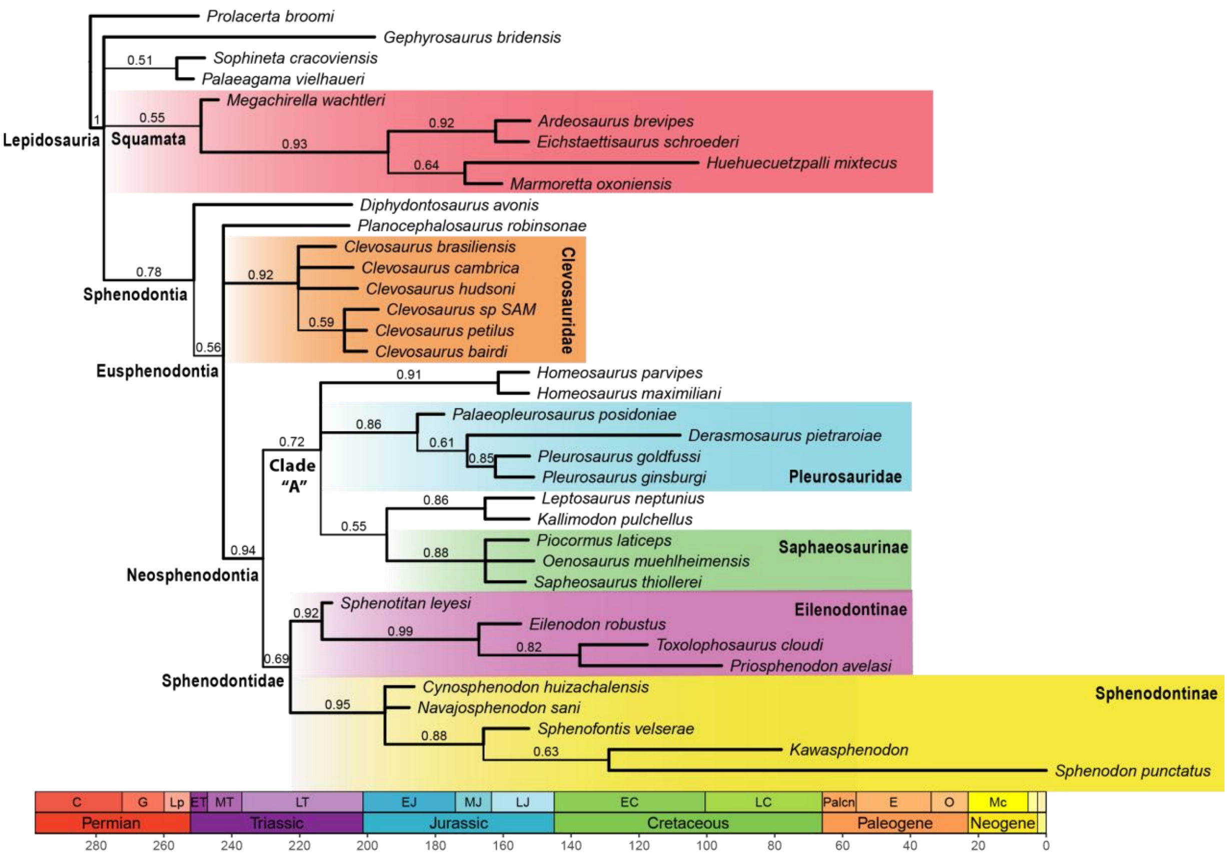

Suppl. Fig. 3. Majority rule consensus tree from the relaxed morphological clock Bayesian inference analysis. Node values indicate posterior probabilities.

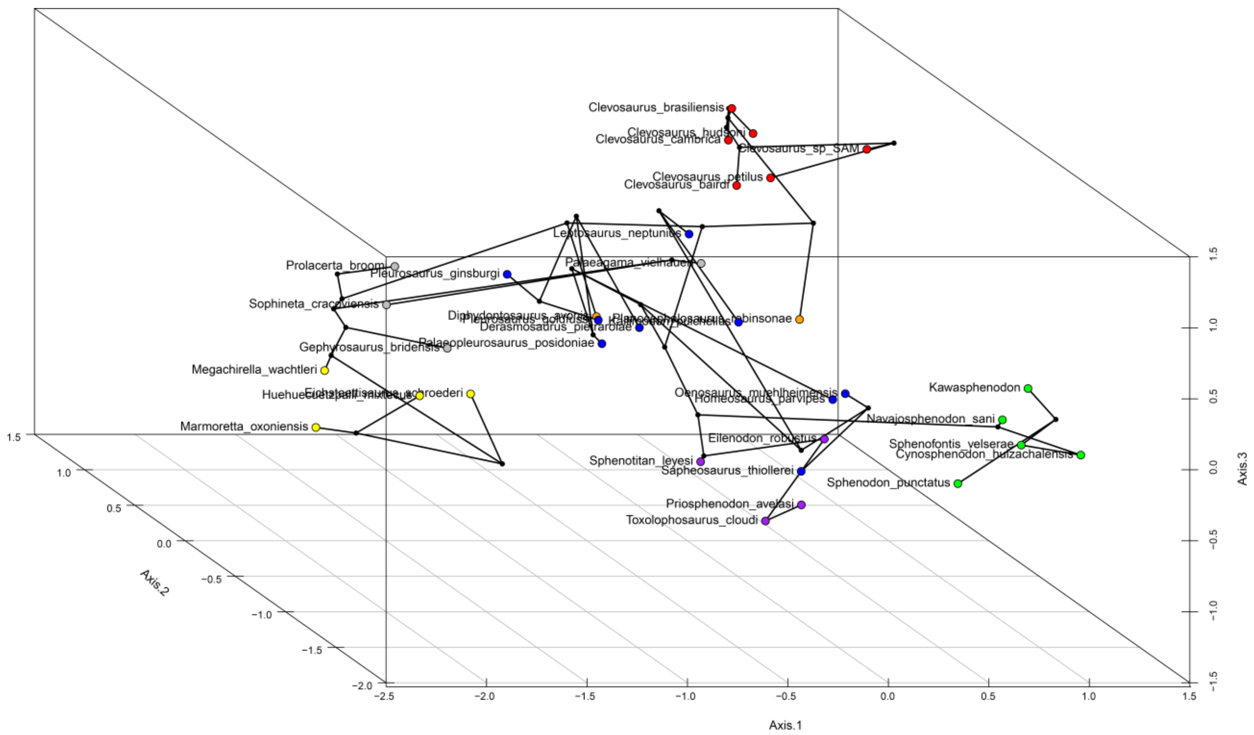

Suppl. Fig. 4. Phylomorphospace of early lepidosaurs and sphenodontians using discrete morphological characters. Clade “A” refers to the clade recovered by the final analysis (relaxed morphological clock Bayesian inference) both here and in Simões *et al.* (2020), including *Homeosaurus*, pleurosaurids, and saphaeosaurids.

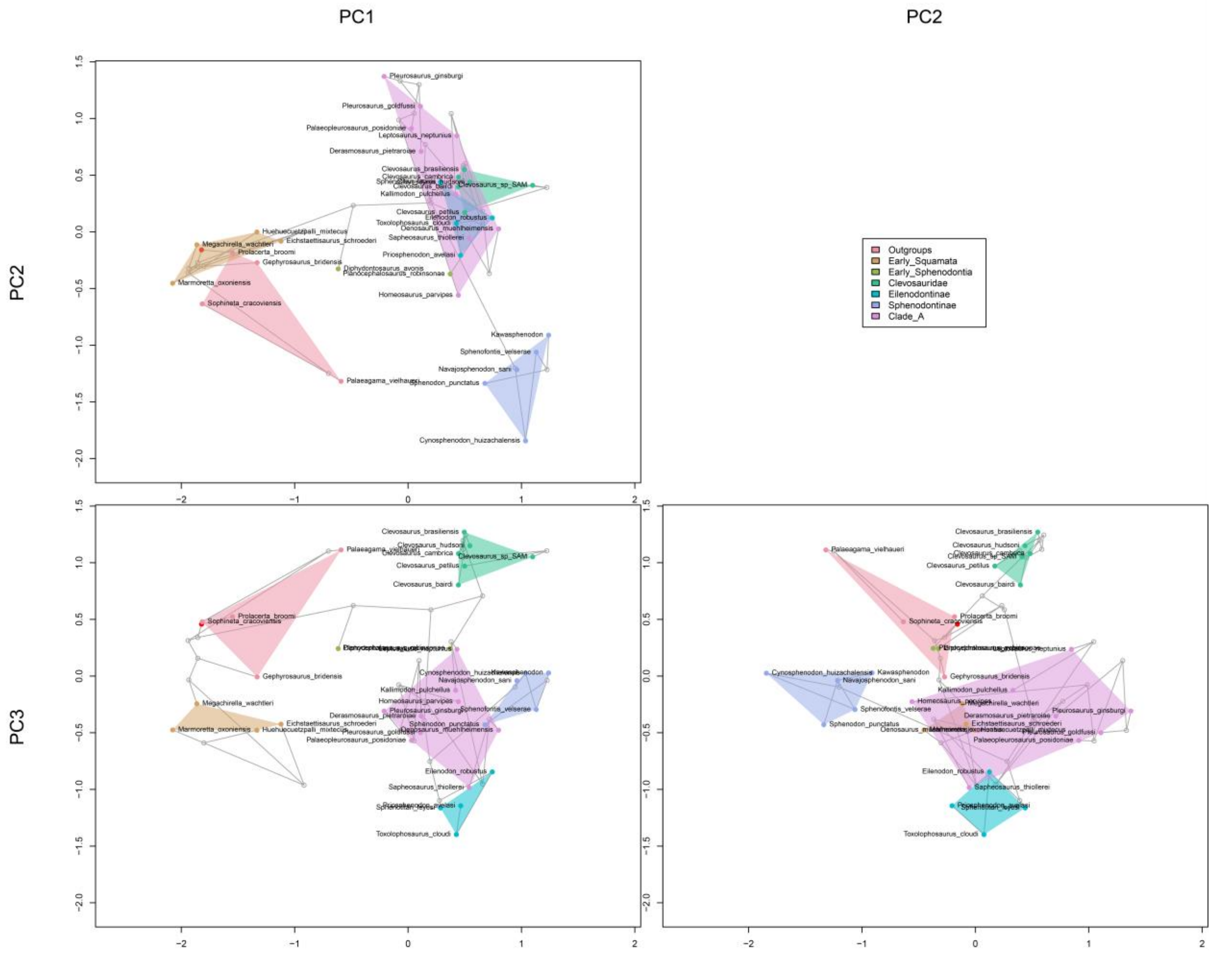

Suppl. Fig. 5. Phylomorphospace of early lepidosaurs and sphenodontians using discrete morphological characters. Principal components 1-3 with textile labels associated with each data point.

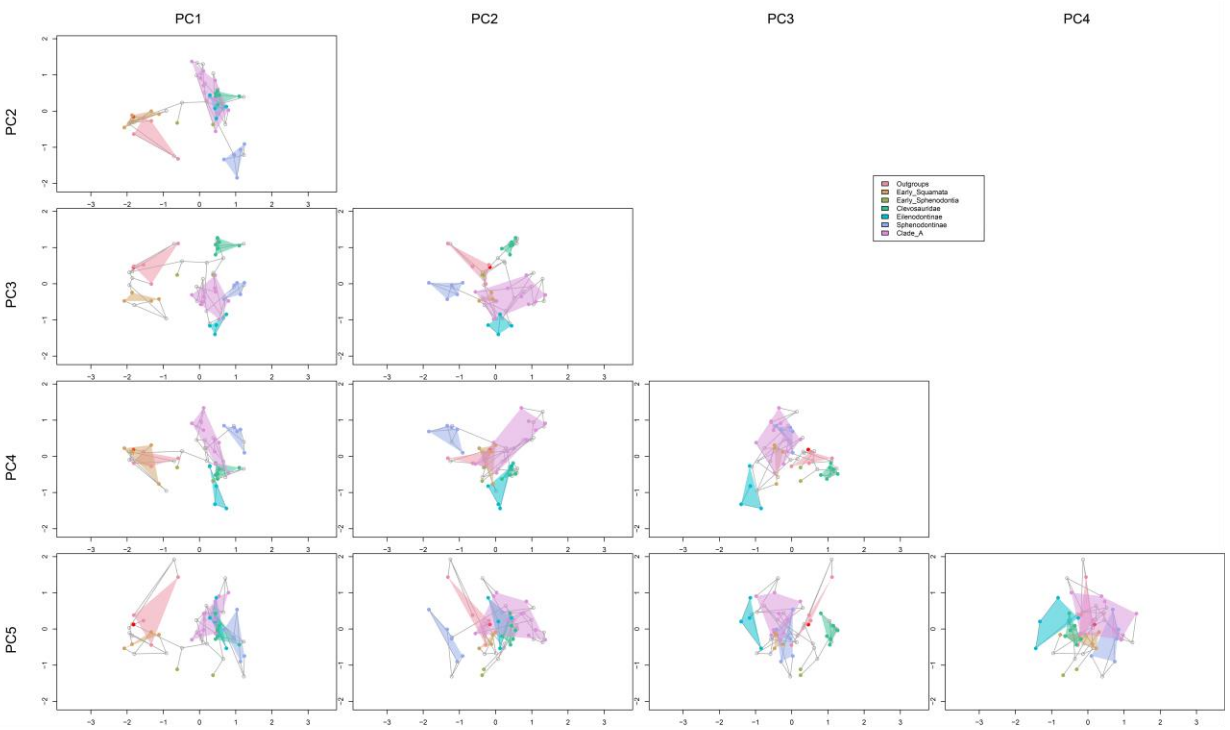

Suppl. Fig. 5. Phylomorphospace of early lepidosaurs and sphenodontians using discrete morphological characters. Principal components 1-5.

Suppl. Table 1. Specimen numbers with discrete ontogenetic markers, length measurement and inferred ontogeny.

Specimen (MCZ)	Elements	Add.teeth (tooth count)	Alt. teeth	Hatch. teeth	Succ. teeth: Anky losis?	DL(mm)	Inferred Ontogeny
MCZ 9093	Dentary + Maxilla	Present (D14+/M10+)	Worn?	?	?	R=17.15+/L= 17.64+	Adult
MCZ 9098	Dentary	Present (D8+/M?)	Absent	?	Yes	16.5+	Adult
MCZ 101562	Dentary	Present (D10+/M?)	?	?	?	11.73+	Adult
MNA.V.8 726	Dentary	Present (D16/M?)	Present	?	Yes	11.7+	Adult
MNA.V.1 2442	Dentary + Maxilla + Palatines	Present (D5/M?)	Present (Worn)	Totally Worn	Yes	R=10.1/L=9. 69	Late juvenile/Sub adult
MCZ 9099	Dentary	Present (D4/M?)	Present	Present	No	6.28	Juvenile
MCZ 101564	Dentary	Present (D2+/M?)	Present	Present	No	6.6	Juvenile
MCZ 101575	Dentary	?	Present	?	No	7.2	Juvenile
MCZ 9094	Dentary + Maxilla + Palatine	Present?	Present	?	?	~7.4	Juvenile
MCZ 9102	Dentary + Maxilla	Present?	Present	?	No	8.5	Juvenile?
MCZ 9103	Dentary + Maxilla	Present (D?/M3+)	Present	?	No	?	Juvenile
MCZ 101569	Dentary + Palatine?	Present (D4/M?)	Present	?	No	~6.13	Juvenile
MCZ 101563	Dentary	Present (D2/M?)	Present	Present	No	6.33	Juvenile
MNA.V.8 727	Dentary	Present (D12/M?)	Present	?	No	6.3	Juvenile
MCZ 9100	Maxilla	Present (D?/M5)	Present (Worn)	?	?	?	?

Abbreviations: Add. teeth, additional teeth; Alt. teeth, alternating teeth; Hatch. teeth, hatchling teeth; Succ. teeth, successional teeth. Tooth count for additional teeth include tooth count for dentary (D) and maxillary teeth (M). Dentary length measure (DL) is taken from the anterior tip of the dentary to the anterior margin of the dentary coronoid process. Numbers followed by (+) indicate minimum tooth count or minimum length (full value unknown due to limited preservation).

Suppl. Table 2. DOIs for all digitized elements described here deposited in MorphoSource.

Media 000394958: Right Squamosal [Mesh] [CT]	https://www.morphosource.org/concern/media/000394958?locale=en	https://doi.org/10.17602/M2/M394958
Media 000392196: Skull And Cervical Vertebrae [CTImageSeries] [CT]	https://www.morphosource.org/concern/media/000392196	https://doi.org/10.17602/M2/M392196
Media 000394967: Right Surangular [Mesh] [CT]	https://www.morphosource.org/concern/media/000394967?locale=en	https://doi.org/10.17602/M2/M394967
Media 000394971: Right Quadrate [Mesh] [CT]	https://www.morphosource.org/concern/media/000394971?locale=en	https://doi.org/10.17602/M2/M394971
Media 000394843: Right Dentary [Mesh] [CT]	https://www.morphosource.org/concern/media/000394843?locale=en	https://doi.org/10.17602/M2/M394843
Media 000394999: Right Palatine [Mesh] [CT]	https://www.morphosource.org/concern/media/000394999?locale=en	https://doi.org/10.17602/M2/M394999
Media 000394766: Nasals [Mesh] [CT]	https://www.morphosource.org/concern/media/000394766?locale=en	https://doi.org/10.17602/M2/M394766
Media 000394867: Left Palatine [Mesh] [CT]	https://www.morphosource.org/concern/media/000394867?locale=en	https://doi.org/10.17602/M2/M394867
Media 000394837: Left Prefrontal [Mesh] [CT]	https://www.morphosource.org/concern/media/000394837?locale=en	https://doi.org/10.17602/M2/M394837
Media 000394993: First Ceratobranchial [Mesh] [CT]	https://www.morphosource.org/concern/media/000394993?locale=en	https://doi.org/10.17602/M2/M394993
Media 000394996: Pterygoids [Mesh] [CT]	https://www.morphosource.org/concern/media/000394996?locale=en	https://doi.org/10.17602/M2/M394996
Media 000394760: Right Maxilla [Mesh] [CT]	https://www.morphosource.org/concern/media/000394760?locale=en	https://doi.org/10.17602/M2/M394760
Media 000394870: Right Prefrontal [Mesh] [CT]	https://www.morphosource.org/concern/media/000394870?locale=en	https://doi.org/10.17602/M2/M394870
Media 000394980: Left Ectopterygoid [Mesh] [CT]	https://www.morphosource.org/concern/media/000394980?locale=en	https://doi.org/10.17602/M2/M394980
Media 000394946: Right Frontal [Mesh] [CT]	https://www.morphosource.org/concern/media/000394946?locale=en	https://doi.org/10.17602/M2/M394946
Media 000394757: Premaxillae [Mesh] [CT]	https://www.morphosource.org/concern/media/000394757?locale=en	https://doi.org/10.17602/M2/M394757
Media 000395041: Neural Arch Of Axis Vertebra [Mesh] [CT]	https://www.morphosource.org/concern/media/000395041?locale=en	https://doi.org/10.17602/M2/M395041
Media 000394846: Right Jugal [Mesh] [CT]	https://www.morphosource.org/concern/media/000394846?locale=en	https://doi.org/10.17602/M2/M394846
Media 000394986: Right Postfrontal [Mesh] [CT]	https://www.morphosource.org/concern/media/000394986?locale=en	https://doi.org/10.17602/M2/M394986
Media 000395021: Atlas Centrum [Mesh] [CT]	https://www.morphosource.org/concern/media/000395021?locale=en	https://doi.org/10.17602/M2/M395021

Media 000394954: Left Parietal [Mesh] [CT]	https://www.morphosource.org/concern/media/000394954?locale=en	https://doi.org/10.17602/M2/M394954
Media 000394857: Left Postorbital [Mesh] [CT]	https://www.morphosource.org/concern/media/000394857?locale=en	https://doi.org/10.17602/M2/M394857
Media 000394977: Left Prearticular [Mesh] [CT]	https://www.morphosource.org/concern/media/000394977?locale=en	https://doi.org/10.17602/M2/M394977
Media 000394964: Left Surangular [Mesh] [CT]	https://www.morphosource.org/concern/media/000394964?locale=en	https://doi.org/10.17602/M2/M394964
Media 000395044: Cervical Vertebrae [Mesh] [CT]	https://www.morphosource.org/concern/media/000395044?locale=en	https://doi.org/10.17602/M2/M395044
Media 000395035: Neural Arch Of Atlas [Mesh] [CT]	https://www.morphosource.org/concern/media/000395035?locale=en	https://doi.org/10.17602/M2/M395035
Media 000395038: Axis Vertebra [Mesh] [CT]	https://www.morphosource.org/concern/media/000395038?locale=en	https://doi.org/10.17602/M2/M395038
Media 000394860: Right Postorbital [Mesh] [CT]	https://www.morphosource.org/concern/media/000394860?locale=en	https://doi.org/10.17602/M2/M394860
Media 000394763: Left Maxilla [Mesh] [CT]	https://www.morphosource.org/concern/media/000394763?locale=en	https://doi.org/10.17602/M2/M394763
Media 000394840: Left Dentary [Mesh] [CT]	https://www.morphosource.org/concern/media/000394840?locale=en	https://doi.org/10.17602/M2/M394840
Media 000394849: Left Jugal [Mesh] [CT]	https://www.morphosource.org/concern/media/000394849?locale=en	https://doi.org/10.17602/M2/M394849
Media 000395018: Atlas Centrum [Mesh] [CT]	https://www.morphosource.org/concern/media/000395018?locale=en	https://doi.org/10.17602/M2/M395018
Media 000394983: Septomaxillae [Mesh] [CT]	https://www.morphosource.org/concern/media/000394983?locale=en	https://doi.org/10.17602/M2/M394983
Media 000394874: Left Frontal [Mesh] [CT]	https://www.morphosource.org/concern/media/000394874?locale=en	https://doi.org/10.17602/M2/M394874
Media 000393255: Right Scapula [Mesh] [CT]	https://www.morphosource.org/concern/media/000393255?locale=en	https://doi.org/10.17602/M2/M393255
Media 000394990: Left Postfrontal [Mesh] [CT]	https://www.morphosource.org/concern/media/000394990?locale=en	https://doi.org/10.17602/M2/M394990
Media 000394961: Left Squamosal [Mesh] [CT]	https://www.morphosource.org/concern/media/000394961?locale=en	https://doi.org/10.17602/M2/M394961
Media 000393231: Right Radius [Mesh] [CT]	https://www.morphosource.org/concern/media/000393231?locale=en	https://doi.org/10.17602/M2/M393231
Media 000393189: Left Humerus [Mesh] [CT]	https://www.morphosource.org/concern/media/000393189?locale=en	https://doi.org/10.17602/M2/M393189
Media 000393284: Right Ulna [Mesh] [CT]	https://www.morphosource.org/concern/media/000393284?locale=en	https://doi.org/10.17602/M2/M393284
Media 000393262: Left Tibia [Mesh] [CT]	https://www.morphosource.org/concern/media/000393262?locale=en	https://doi.org/10.17602/M2/M393262
Media 000393195: Right Humerus [Mesh] [CT]	https://www.morphosource.org/concern/media/000393195?locale=en	https://doi.org/10.17602/M2/M393195
Media 000392044: Right Coracoid [Mesh] [CT]	https://www.morphosource.org/concern/media/000392044?locale=en	https://doi.org/10.17602/M2/M392044

Media 000393265: Left Ulna [Mesh] [CT]	https://www.morphosource.org/concern/media/000393265?locale=en	https://doi.org/10.17602/M2/M393265
Media 000392923: Left Fibula [Mesh] [CT]	https://www.morphosource.org/concern/media/000392923?locale=en	https://doi.org/10.17602/M2/M392923
Media 000393207: Pelvic Girdle [Mesh] [CT]	https://www.morphosource.org/concern/media/000393207?locale=en	https://doi.org/10.17602/M2/M393207
Media 000392932: Right Fibula [Mesh] [CT]	https://www.morphosource.org/concern/media/000392932?locale=en	https://doi.org/10.17602/M2/M392932
Media 000391984: Caudal Vertebrae [Mesh] [CT]	https://www.morphosource.org/concern/media/000391984?locale=en	https://doi.org/10.17602/M2/M391984
Media 000392047: Dorsal Vertebrae [Mesh] [CT]	https://www.morphosource.org/concern/media/000392047?locale=en	https://doi.org/10.17602/M2/M392047
Media 000392050: Left Femur [Mesh] [CT]	https://www.morphosource.org/concern/media/000392050?locale=en	https://doi.org/10.17602/M2/M392050
Media 000393252: Left Scapula [Mesh] [CT]	https://www.morphosource.org/concern/media/000393252?locale=en	https://doi.org/10.17602/M2/M393252
Media 000393243: Ribs [Mesh] [CT]	https://www.morphosource.org/concern/media/000393243?locale=en	https://doi.org/10.17602/M2/M393243
Media 000391921: Skeleton [CTImageSeries] [CT]	https://www.morphosource.org/concern/media/000391921	https://doi.org/10.17602/M2/M391921
Media 000392010: Left Coracoid [Mesh] [CT]	https://www.morphosource.org/concern/media/000392010?locale=en	https://doi.org/10.17602/M2/M392010
Media 000393217: Left Radius [Mesh] [CT]	https://www.morphosource.org/concern/media/000393217?locale=en	https://doi.org/10.17602/M2/M393217

Supplementary references

Simões, T. R., Caldwell, M. W., Pierce, S. E. 2020. Sphenodontian phylogeny and the impact of model choice in bayesian morphological clock estimates of divergence times and evolutionary rates. BMC Biol. 18, 191.
