# Supplementary material for: An exceptionally preserved Sphenodon-like sphenodontian reveals deep time conservation of the tuatara skeleton and ontogeny
Source: Commun Biol. 2022 Mar 3;5:195. doi: 10.1038/s42003-022-03144-y (PMC8894340; doi:10.1038/s42003-022-03144-y)
Supplement: Supplementary file 3 — Reporting Summary [file 42003_2022_3144_MOESM3_ESM.pdf]

nature portfolio

Corresponding author(s): Tiago R. Simões

Last updated by author(s): Jan 14, 2022

Reporting Summary

Nature Portfolio wishes to improve the reproducibility of the work that we publish. This form provides structure for consistency and transparency in reporting. For further information on Nature Portfolio policies, see our Editorial Policies and the Editorial Policy Checklist.

Statistics

For all statistical analyses, confirm that the following items are present in the figure legend, table legend, main text, or Methods section.

n/a	Confirmed	
☐	☒	The exact sample size (*n*) for each experimental group/condition, given as a discrete number and unit of measurement
☒	☐	A statement on whether measurements were taken from distinct samples or whether the same sample was measured repeatedly
☒	☐	The statistical test(s) used AND whether they are one- or two-sided<br>*Only common tests should be described solely by name; describe more complex techniques in the Methods section.*
☒	☐	A description of all covariates tested
☒	☐	A description of any assumptions or corrections, such as tests of normality and adjustment for multiple comparisons
☒	☐	A full description of the statistical parameters including central tendency (e.g. means) or other basic estimates (e.g. regression coefficient) AND variation (e.g. standard deviation) or associated estimates of uncertainty (e.g. confidence intervals)
☒	☐	For null hypothesis testing, the test statistic (e.g. *F*, *t*, *r*) with confidence intervals, effect sizes, degrees of freedom and *P* value noted<br>*Give P values as exact values whenever suitable.*
☐	☒	For Bayesian analysis, information on the choice of priors and Markov chain Monte Carlo settings
☒	☐	For hierarchical and complex designs, identification of the appropriate level for tests and full reporting of outcomes
☒	☐	Estimates of effect sizes (e.g. Cohen's *d*, Pearson's *r*), indicating how they were calculated

Our web collection on statistics for biologists contains articles on many of the points above.

Software and code

Policy information about availability of computer code

Data collection	No software was used for data collection
Data analysis	Our data was compiled in Mesquite (v. 3.04); our analysis were performed in the phylogenetic software TNT (v 1.5) and Mr. Bayes (v. 3.2.7a). Morphospace analyses and graphic plots were conducted/produced in R. All the necessary code for phylogenetic analyses and R scripts are provided as supplementary data.

For manuscripts utilizing custom algorithms or software that are central to the research but not yet described in published literature, software must be made available to editors and reviewers. We strongly encourage code deposition in a community repository (e.g. GitHub). See the Nature Portfolio guidelines for submitting code & software for further information.

Data

Policy information about availability of data

All manuscripts must include a data availability statement. This statement should provide the following information, where applicable:

- Accession codes, unique identifiers, or web links for publicly available datasets
- A description of any restrictions on data availability
- For clinical datasets or third party data, please ensure that the statement adheres to our policy

All data sets, tree files, log files and script files are available on Harvard Dataverse (permanent link provided in the paper). All CT scan data is available online in MorphoSource.org (links provided in the main text and supplementary information)

Field-specific reporting

Please select the one below that is the best fit for your research. If you are not sure, read the appropriate sections before making your selection.

☐ Life sciences ☐ Behavioural & social sciences ☒ Ecological, evolutionary & environmental sciences

For a reference copy of the document with all sections, see nature.com/documents/nr-reporting-summary-flat.pdf

Ecological, evolutionary & environmental sciences study design

All studies must disclose on these points even when the disclosure is negative.

Study description	We present a detailed morphological and phylogenetic assessment of new species of sphenodontian reptiles from the Early Jurassic of the United States. Using CT scan data and time calibrated bayesian inference analysis we reveal several traits that have long been assumed to be exclusive to the single living member of sphenodontians to have originated at least 190 million years ago.
Research sample	The data includes discrete morphological characters obtained from 36 species representing all major groups of sphenodontian reptiles. Most of the original data comes from a previous study [ref. 19], with the addition of new anatomical information from the new species described here
Sampling strategy	All new specimens described here were collected in the 1982 field season by a team led by Dr. Farish Jenkins (Harvard University) in the state of Arizona, United States. See also ref 19 for sampling of other species included in phylogenetic analysis
Data collection	Anatomical data from the specimens described here were obtained through direct personal observation (using dissecting microscopes), photographs, and micro-CT scanning: X-Tek Micro-CT scanner and Xradia Versa 620 Zeiss micro-CT scanner.
Timing and spatial scale	N/A
Data exclusions	No data were excluded
Reproducibility	All data necessary to verify our observations have been deposited online (see data availability statement) and all necessary input data files to reproduce analytical protocols have also been deposited online (see code availability statement).
Randomization	N/A
Blinding	Blinding was not relevant to this data set, and set of analyses.

Did the study involve field work? ☐ Yes ☒ No

Reporting for specific materials, systems and methods

We require information from authors about some types of materials, experimental systems and methods used in many studies. Here, indicate whether each material, system or method listed is relevant to your study. If you are not sure if a list item applies to your research, read the appropriate section before selecting a response.

Materials & experimental systems

n/a	Involved in the study	
☒	☐	Antibodies
☒	☐	Eukaryotic cell lines
☐	☒	Palaeontology and archaeology
☒	☐	Animals and other organisms
☒	☐	Human research participants
☒	☐	Clinical data
☒	☐	Dual use research of concern

Methods

n/a	Involved in the study	
☒	☐	ChIP-seq
☒	☐	Flow cytometry
☒	☐	MRI-based neuroimaging

Palaeontology and Archaeology

Specimen provenance	The fossils were collected under permit No. 82-AZ-139, in direct charge of F.A. Jenkins, Jr. and C.R. Schaff. Field work on the Navajo Nation was conducted under a permit from the Navajo Nation Minerals Department. Any persons wishing to conduct geologic investigations on the Navajo Nation must first apply for, and receive, a permit from the Navajo Nation Minerals Department, P.O. Box 1910, Window Rock, Arizona 86515, and telephone # (928) 871-6588.
Specimen deposition	All specimens are deposited on publicly accessible natural history collections: the Museum of Comparative Zoology (Harvard University) and Museum of Northern Arizona.
Dating methods	No new dates were provided for individual specimens.

☒ Tick this box to confirm that the raw and calibrated dates are available in the paper or in Supplementary Information.

Ethics oversight	The results of this study have been shared and approved by representatives of the Navajo Nation. Any persons wishing to conduct geologic investigations on the Navajo Nation must first apply for, and receive, a permit from the Navajo Nation Minerals Department, P.O. Box 1910, Window Rock, Arizona 86515, and telephone # (928) 871-6588.

Note that full information on the approval of the study protocol must also be provided in the manuscript.
